# Participatory Epidemiology of Major Camel Diseases and Their Management in Somalia: The Case of Banadir and Lower Shabelle Regions

**DOI:** 10.1155/vmi/3560931

**Published:** 2026-10-25

**Authors:** Omar Salah, Yasin Sh Hassan, Mohamed Yusuf, Hassan AbdiNB

**Affiliations:** ^1^ Faculty of Veterinary Medicine and Animal Husbandry Somali National University (SNU) Mogadishu Somalia; ^2^ Department of Veterinary Medicine Faculty of Veterinary Medicine and Animal Husbandry University of Somalia (UNISO) Mogadishu Somalia; ^3^ Department of Animal Production Ministry of Livestock, Forestry and Range Mogadishu Somalia

**Keywords:** anthrax, camel, disease impact, disease indicators, indigenous knowledge, matrix scoring, traditional treatment, trypanosomosis

## Abstract

This study investigates major camel diseases, their community‐perceived epidemiological characteristics, and management practices in the Banadir and Lower Shabelle regions of Somalia. Data were collected from 17 key informants through semistructured interviews and from 18 participants across three focus group discussions (FGDs). Semistructured interviews captured key camel diseases, their local names, disease impacts, coping mechanisms, and locally used disease management approaches, while FGDs were used to conduct participatory exercises to rank and characterize diseases based on severity and clinical signs. Kendall’s W was used to assess consensus among participants, revealing the strength of the agreement across groups. Ten diseases were most frequently reported, including trypanosomosis, mastitis, pneumonia, ringworm, camelpox, mange, tetanus, wry neck, parasite infection, and anthrax. For disease management practices, about 15 out of 17 respondents reported using both modern medicines and traditional practices such as burning, administering *Dobera glabra* seeds, and applying camel hump ghee. In addition, 13 out of 17 informants indicated that farmers do not conduct regular animal health checkups. Preventive measures included isolation, relocation, and recitation of the Holy Quran. Regarding veterinary consultation, 10 out of 17 respondents reported poor veterinary consultation, citing limited awareness and poor accessibility as potential causes. Of the eight diseases assessed by pairwise ranking, farmers rated anthrax (Kud) as the most severe (mean 6.67 ± 0.58 out of 7), followed by tetanus (Taagow, 6.00 ± 1.00), with strong, statistically significant agreement across the focus groups (Kendall’s *W* = 0.849; *p* = 0.013). Wry neck and trypanosomosis were rated moderately severe, while pneumonia, mange, mastitis, and camelpox scored lowest.

Matrix scoring showed strong, statistically significant agreement among the three informant groups across all disease signs (Kendall’s *W* = 0.74–1.00; *p* < 0.05). The strongest associations were dyspnea and nasal discharge with pneumonia (mean scores 15.00 ± 1.00/20 and 16.67 ± 2.08/20), odor change with trypanosomosis (15.00 ± 3.00/20), and fever with camelpox and trypanosomosis.

The findings also revealed clear seasonal variation in camel diseases, with most conditions peaking during the rainy seasons of spring (Gu) and autumn (Dayr). In contrast, trypanosomosis, tetanus, and mange occurred throughout the year, while anthrax was predominantly associated with the dry Jiilaal season. Major challenges reported include limited veterinary services, high treatment costs, and inadequate feed and drug supplies. These findings reflect substantial indigenous knowledge and point to the need for integrated interventions including improved veterinary services, affordable drugs and vaccines, and farmer capacity building to strengthen camel health in the region.

## 1. Introduction

Camels are uniquely adapted to survive in harsh, arid regions of the world and capable of enduring extreme dehydration and efficiently conserving water through specialized physiological traits. This adaptation enables them to thrive in environments inhospitable to other livestock and makes them invaluable to communities for transportation, milk, and meat [1].

In Somali society, camels are primarily valued for their meat and milk production. On the other hand, male camels are used for transporting water and household items when families relocate within their range [2]. Camels also hold significant economic value within the pastoral system, acting as a financial safeguard against the harsh impacts of droughts and diseases that more severely affect smaller livestock. Moreover, camels hold a place of cultural importance in Somali society, as evidenced by their frequent mentions in Somali oral literature [3]. In times of hardship and livestock losses, camels are provided by family networks to support affected households [4].

Diseases are among the primary constraints in camel husbandry, thus affecting the livelihoods of communities that depend on them. Diseases significantly affect camel productivity, causing high mortality and reducing camels’ productive lifespans as conditions such as brucellosis can lower birth rates, affecting the economic viability of camel farming [5]. Additionally, diseases incur direct costs for veterinary care and medication and may necessitate culling infected animals. Some camel diseases also pose zoonotic risks, threatening human health and emphasizing the need for integrated approaches to managing camel health that combine veterinary and public health strategies [6].

Significant diseases impacting dromedary camels economically include camelpox, contagious ecthyma, rabies, pasteurellosis, leptospirosis, *Q* fever, trypanosomosis, mange, dermatophytosis, gastrointestinal parasites, and tick infestation. These diseases are major concerns for camel health and productivity [7].

In Somalia, camel diseases were reported as early as the colonial era in 1938. Since then, many diseases have been identified and documented, including anthrax [8], trypanosomiasis [9], camelpox [10], brucellosis [11], hemorrhagic septicemia [12], papillomatosis [13], and mastitis [14]. Although some of these studies are current, many are outdated and many do not focus on disease characteristics, impact, and importance. There are no documented data on camel disease knowledge and management practices, highlighting a significant gap that must be addressed for effective disease management.

Therefore, this study aims to identify community‐perceived epidemiology and management of common camel diseases in the Banadir and Lower Shabelle regions of Somalia.

## 2. Materials and Methods

### 2.1. Description of the Study Area

This study was conducted in Banadir and Lower Shabelle regions of southern Somalia as illustrated in Figure 1. Banadir, centered in Mogadishu, has a coastal climate with high humidity and saline air at 2.0469 N, 45.3182 E. Lower Shabelle, from 1.9167 N, 44.7833 E, features diverse landscapes, including riverine, agricultural, and arid zones, representing pastoral and agropastoral settings. The study area experiences temperatures ranging from 20°C to 30°C during the rainy season (April–June, October–November) and 30°C to 40°C in the dry months (January–March, July–September) [15]. The study area experiences two rainy seasons, Spring (Gu) season from April to June and Autumn (Dayr) from October to December, and the annual rainfall typically totals between 400 and 600 mm, [15]. The combined total population is around half a million camels [16]. This study was particularly conducted in high camel population districts, including Dayniile and Hodan in Banadir, and Afgooye in Lower Shabelle, as shown in the map in Figure 1.

**FIGURE 1:**
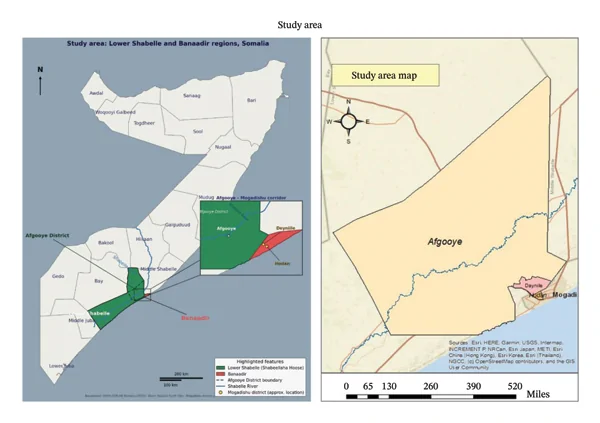
Map of the study area showing the location of the Banadir and Lower Shabelle regions within Somalia (left) and the three selected study districts—Afgooye, Hodan, and Dayniile (right).

### 2.2. Study Population and Design

The study population includes camel herders, owners, dairy farm managers, veterinary doctors, and community elders with camel‐rearing experience. A cross‐sectional study was conducted from July to October 2024, using a participatory epidemiological approach to gather detailed information on major camel diseases, their occurrence patterns, and management practices.

To select participants for the study, a multistage purposive sampling method was employed. Initially, five camel farming areas (subdistrict administrative units) were judgmentally chosen from each of the three districts. From these, one area was randomly selected in each district. Subsequently, six participants from each of these selected areas were chosen, resulting in a total of 18 participants for the focus group discussions (FGDs). The number of participants was considered appropriate for participatory epidemiological exercises, as it facilitated active engagement, effective group interaction, and consensus building during discussions. The number of FGDs was determined based on qualitative saturation principles. Given the homogeneous nature of the study population and the use of a semistructured discussion guide, three FGDs were considered sufficient to capture the main themes related to camel diseases and associated clinical indicators. Previous methodological evidence indicates that three to six FGDs can achieve saturation in homogeneous populations, with three groups being sufficient to identify the most prevalent themes [17, 18].

For the semistructured interviews, a purposive sample of 17 key informant interview participants was selected, including five participants from each of Hodan and Dayniile districts in the Banadir region and seven from Afgooye district in the Lower Shabelle region. A slightly larger sample was allocated to Afgooye to capture perspectives from a different region. Participant selection was guided by stakeholder diversity, district coverage, field feasibility, and the need to obtain detailed local knowledge on camel diseases and management practices. These considerations informed the relatively small sample size, which was appropriate for generating in‐depth qualitative insights. This approach aligns with the methodologies supported by other researchers, which emphasize that saturation with data from in‐depth interviews can be reached under 25 interviews, more specifically between 9 and 17 interviews [19].

### 2.3. Methods of Data Collection

Participatory data collection methods were utilized, including semistructured interviews and FGDs. Semistructured interviews were used to identify key camel diseases, their local names, disease impacts, coping mechanisms, and locally used disease management approaches. Interviews lasted approximately 30–60 min, were conducted in Somali, audio‐recorded with participants’ consent, and subsequently transcribed. The interview guide was pretested before the main data collection to assess its clarity and relevance.

FGDs were conducted in Somali, with each session lasting approximately 90–120 min. Across three FGDs, pairwise comparison techniques were applied to assess the relative importance and perceived impact of identified diseases. Matrix scoring was also employed during FGDs to evaluate the association between previously identified diseases and clinical signs. In this exercise, diseases were listed along the *x*‐axis and clinical indicators on the *y*‐axis. Participants were provided with a fixed total of 20 counters (stones) and were instructed by the facilitators to distribute them across diseases for each clinical sign at a time. More counters were allocated to diseases perceived as being more strongly associated with a given clinical sign, while fewer or no counters were assigned to weakly associated or unrelated diseases. This ensured a proportional representation of perceived disease–sign relationships.

Disease scoring was based on group consensus within each FGD rather than individual scoring. The outputs from the three FGDs were then aggregated and analyzed to compute mean ± standard deviation values using SPSS. This aggregation was performed at the group level, treating each FGD as a single analytical unit to ensure consistency in interpretation across discussion groups. Of the 10 most frequently reported diseases, eight were selected for pairwise ranking. Parasite infection was excluded as a broad nonspecific category encompassing multiple agents with variable presentations; ringworm was excluded to preserve the discriminatory power of pairwise comparison across the remaining conditions.

During data collection, informed consent was obtained in the form of signed form from all participants including key informants and in the FGDs. Participants were guaranteed the confidentiality of their personal information and identity.

### 2.4. Data Analysis Methods

Data collected in the study were initially recorded in Microsoft Excel and then transferred to SPSS Statistics Version 20 for analysis. Descriptive statistical methods, including counts, frequencies, means, and standard deviations, were used to summarize respondents’ characteristics, disease occurrence, disease management practices, and matrix scoring results.

Consensus‐based responses obtained from FGDs were analyzed to assess the level of agreement among participants regarding the relative importance, characteristics, and clinical indicators of camel diseases. Mean scores were calculated from the aggregated focus group results to summarize disease rankings and perceived disease–indicator associations.

Inferential statistical analyses were also conducted where appropriate. The Friedman test was applied to appropriately analyze the ranked disease severity scores across the eight diseases. Spearman’s rank correlation was applied to examine associations between ranked variables, including disease mean ranks and frequency‐related measures. Statistical significance was assessed at an alpha level of 0.05, and exact *p* values were reported.

### 2.5. Limitations of the Study

This study has several limitations. Farmers’ recall of experiences over several years introduces potential recall bias, and all disease identifications rest on community perception rather than clinical or laboratory confirmation, so locally recognized syndromes may not correspond exactly to confirmed etiologies and conditions with overlapping signs are liable to be conflated. In addition, the concordance analysis was based on only three informant groups and the perception–frequency correlation on only eight diseases, limiting statistical power.

## 3. RESULTS

A total of 17 key informants participated in the semistructured interviews. The majority of the informants were male (16), and their ages averaged 43 years (minimum 28, maximum 70). In terms of education, six of the informants had informal education, five held a bachelor’s degree, four a master’s degree, one a PhD, and one had completed high school. Regarding occupation, eight were farmers, while the remaining informants were veterinary doctors working as academics (3), private veterinarians (3), and government officers (3). Their years of experience were distributed as 10 years or less (9) and more than 10 years (8) (Table 1).

**TABLE 1:** Demographic characteristics of the key informants (*n* = 17).

Variable	Level	Number
Gender	Male	16
	Female	1

Age	18–34 years	6
	35–64 years	8
	65+ years	3

Education level	Informal education	6
	High school	1
	Bachelor’s degree	5
	Master’s degree	4
	PhD	1

Occupation	Farmer	8
	Private veterinarian	3
	Government officer	3
	Academic	3

Years of experience^∗^	≤ 10 Years	9
	> 10 Years	8

^∗^The term “years of experience” refers to the total number of years each informant has been engaged in their respective professional activities. This includes veterinarians working in government institutions, academic settings, or private practice, as well as camel farmers involved in camel husbandry. Values are descriptive counts of purposively selected key informants.

### 3.1. Thematic Analysis of Semistructured Interview

#### 3.1.1. Common Diseases

Informants were asked to mention the most common diseases in the area for the past 2 years; 10 of most mentioned diseases in descending order include mastitis, trypanosomosis, pneumonia, ringworm, camelpox, mange, tetanus, wry neck, parasite infection, and anthrax.

#### 3.1.2. Disease Management

Analysis of the semistructured interviews revealed that 15 of the 17 respondents stated that farmers primarily rely on treatment with modern medicine for disease management followed by traditional medicines and isolation, while vaccination and proper hygiene strategies were less frequently mentioned.

Informants noted several traditional methods for disease management in the study area. The most popular method is burning, followed by administering soup made from goat meat, seeds from the *Dobera glabra* tree (Garas), *aloe vera* leaves, and camel hump ghee. Other less commonly practiced methods include bloodletting, fasting the animals, and reciting the Holy Quran.

Informants described several common disease prevention methods practiced in the study area. These include relocating animals away from areas with disease outbreaks, isolating and quarantining affected animals, and reciting the Holy Quran over the animals.

Informants revealed that the most frequently used drugs in the study area include oxytetracycline, Pen‐strep, albendazole, and ivermectin. Other drugs mentioned were sulfadiazine, Triquin, and homidium chloride. Additionally, cypermethrin was identified as the most used type of acaricide.

#### 3.1.3. Consulting Veterinarian and Health Checkups

Most informants (10 out of 17) suggested that camel farmers typically rely on their own experience and the advice of fellow farmers rather than consulting with veterinarians. One informant attributed this lack of professional consultation to a general lack of awareness and a belief that veterinarians would be of little help. Additionally, about 13 of the 17 informants reported that farmers do not conduct regular health checkups on their animals. In contrast, the remainder noted that farmers engage in daily self‐observations to monitor their animals’ health.

#### 3.1.4. Impacts of Disease and Challenges Faced by Camel Farmers

When discussing the impacts of diseases, informants reported a variety of consequences including reduced milk production, reproductive problems, and animal death. Other effects noted were market loss, loss of body weight and growth retardation, and high treatment costs. Less commonly, they mentioned owner aggression and mental health issues.

Informants described several coping mechanisms to mitigate the impacts of camel diseases. These include selling healthy stock to generate income, diversifying into other businesses or relying on different animal species owned by the farmer, and seeking loans or assistance from relatives and friends. Additionally, to reduce production costs, some farmers send their animals to graze on rangeland to save on feeding expenses.

Informants reported that camel farmers encounter several significant challenges. Predominantly, disease and shortage of veterinary services pose serious issues. Other critical challenges include the farmers’ limited knowledge and skills, as well as shortages of quality feed and drugs. As per the informants, camel farmers also face poor public infrastructure, such as inadequate roads. Moreover, issues such as conflict and insecurity, insufficient housing and space, and poor government support were mentioned to further complicate their situation.

### 3.2. Pairwise Ranking of Disease Severity

Regarding perceived disease severity, anthrax (Kud) was ranked as the most severe camel disease, with the highest mean score of 6.67 ± 0.58 and a range of 6–7, followed by tetanus (Taagow) with a mean score of 6.00 ± 1.00 and a range of 5–7. Wry neck (Shimbir) and trypanosomosis (Dhukaan) were perceived as moderately severe, scoring 4.33 ± 1.15 and 4.00 ± 0.00, with ranges of 3–5 and 4–4, respectively. Lower severity scores were recorded for pneumonia (Dhugato), mange (Cadho), mastitis (Candho barar), and camelpox (Furuq), with mean scores ranging from 1.33 to 2.33, suggesting relatively lower perceived severity among the focus group participants. A Friedman test confirmed [significant/nonsignificant] differences in perceived severity across the eight diseases (*χ* ^2^ ([7]) = [17.823], *p* = [0.013]). The detailed results of pairwise ranking are presented in Table 2.

**TABLE 2:** Pairwise ranking of camel diseases according to perceived impact and severity (*k* = 3 focus groups).

Disease	Local name	Mean win‐score ± SD (0–7)	Min–max
Anthrax	*Kud*	6.67 ± 0.58^a^	6–7^a^
Tetanus	*Taagow*	6.00 ± 1.00	5–7
Wry neck	*Shimbir*	4.33 ± 1.15	3–5
Trypanosomosis	*Dhukaan*	4.00 ± 0.00	4–4
Pneumonia	*Dhugato*	2.33 ± 1.15	1–3
Mange	*Cadho*	2.00 ± 1.00	1–3
Mastitis	*Candho barar*	1.67 ± 1.15	1–3
Camelpox	*Furuq*	1.33 ± 1.15	0–2^b^

*Note:* Scoring: number of wins across 28 pairwise comparisons per group; possible range 0–7. *k*: number of focus group discussions, each group consists of 6 participants. Kendall’s coefficient of concordance *W* = 0.849; *χ* ^2^ (7) = 17.823; *p* = 0.013. *W* = 1 denotes perfect agreement; values near 1 indicate strong concordance among the three groups. No tied‐rank correction was required.

^a^Highest mean win score.

^b^Lowest mean win score.

### 3.3. Matrix Scoring of Disease Indicators and Seasonality

Matrix scoring was carried out through three FGDs held in Afgooye, Dayniile, and Hodan districts. In these sessions, participants were given 20 counters each for various indicators and asked to distribute these among eight diseases, reflecting the strength of association between specific clinical signs and each disease. The detailed results of this matrix scoring, which evaluates the common camel diseases in the study area, are presented in Table 3.

**TABLE 3:** Matrix scoring for disease indicators.

Indicator	*W*	*χ* ^2^ (df = 7)	*p* value	Mean score of diseases (mean ± SD); *N* = 10 indicators, *k* = 3 informant groups, *n* = 8 diseases							
				Trypanosomosis	Pneumonia	Tetanus	Wry neck	Camelpox	Anthrax	Mange	Mastitis
Fever	0.83^∗^	17.43	0.015	6 ± 1	2 ± 0	0 ± 0	1.33 ± 1.52	7.33 ± 1.15	2.33 ± 0.57	0 ± 0	1 ± 1.73
Nasal discharge	0.93^∗^	19.53	0.007	0.33 ± 0.57	16.67 ± 2.08	0 ± 0	0 ± 0	3 ± 1.73	0 ± 0	0 ± 0	0 ± 0
Reduced milk production	0.82^∗^	17.22	0.016	2.67 ± 2.51	2.33 ± 0.57	0 ± 0	0.67 ± 1.15	3.33 ± 0.57	0 ± 0	1 ± 1	10 ± 2
Dyspnea	0.81^∗^	17.01	0.017	0 ± 0	15 ± 1	3 ± 1	1.33 ± 2.30	0 ± 0	0.67 ± 1.15	0 ± 0	0 ± 0
Paralysis	1^∗^ ^,^ ^†^	21.00	0.004	0 ± 0	0 ± 0	13.67 ± 1.52	6.33 ± 1.52	0 ± 0	0 ± 0	0 ± 0	0 ± 0
Skin lesions	0.93^∗^	19.53	0.007	0.33 ± 0.57	0 ± 0	0 ± 0	0 ± 0	9.67 ± 0.57	0 ± 0	10 ± 0	0 ± 0
Stiffness	0.98^∗^	20.58	0.004	0 ± 0	0 ± 0	9.67 ± 1.52	10.33 ± 1.52	0 ± 0	0 ± 0	0 ± 0	0 ± 0
Odor change	0.74^∗^	15.54	0.030	15 ± 3	1.33 ± 1.15	0 ± 0	0 ± 0	0.33 ± 0.57	1.33 ± 1.15	1.67 ± 1.52	0.33 ± 0.57
Emaciation	0.91^∗^	19.11	0.008	7.67 ± 2.08	4.67 ± 0.57	0 ± 0	0.67 ± 1.15	2.33 ± 1.52	0 ± 0	4.33 ± 0.57	0.33 ± 0.57
Sudden death	0.9^∗^	18.90	0.009	0 ± 0	0.67 ± 0.57	8 ± 2	0 ± 0	0.67 ± 0.57	10.67 ± 2.3	0 ± 0	0 ± 0

*Note: k*: number of informant groups; *n*: number of diseases assessed; *N*: number of clinical indicators assessed. *W*: Kendall’s coefficient of concordance (*W* = 1 indicates perfect agreement among groups; values closer to 1 indicate stronger concordance).

^∗^Statistically significant agreement.

^†^ *W* = 1.000 may reflect a ceiling effect given *k* = 3 groups; therefore, this result should be interpreted with caution. Scoring was performed out of 20 counters distributed across the diseases for each sign; the higher the score, the more strongly pastoralists associated that clinical sign with the given disease. SD values are computed across *k* = 3 FGD groups (df = 2) and should be interpreted with caution.

The analysis of the matrix scoring indicated a high level of agreement across the three informant groups for all disease signs, with all indicators showing statistically significant concordance, with Kendall’s *W* ranging from 0.74 to 1.00 and *p* values < 0.05. This highlights a robust consensus among the informants. Notably, dyspnea and nasal discharge were strongly linked to pneumonia, receiving mean scores of 15.00 ± 1 and 16.67 ± 2.08, respectively. Odor change, receiving a high score of 15 ± 3, was predominantly associated with trypanosomosis.

Fever was associated with both camelpox and trypanosomosis with a mean score of 7.33 ± 1.15 and 6 ± 1 respectively. On the other hand, trypanosomosis was also associated with emaciation with a mean score of 7.67 ± 2.08.

Paralysis and stiffness were clearly attributed to tetanus and wry neck, with significant mean scores of 13.67 ± 1.52 for tetanus and 9.67 ± 1.52 along with 10.33 ± 1.52 for wry neck. Furthermore, a notable association was observed between reduced milk production and mastitis, which scored 10 ± 2. Finally, sudden death was closely associated with both anthrax and tetanus, evidenced by scores of 10.67 ± 2.3 and 8 ± 2, respectively, indicating the critical severity of these diseases as perceived by the community.

Results of FGDs also showed clear seasonal variation in camel diseases. Most diseases peak during rainy seasons, especially Gu and Dayr. Pneumonia is highest in Gu (11.67 ± 2.00), while camelpox (8.67 ± 1.15) and mastitis (7.67 ± 0.58) peak in Gu and remain high in Dayr (8.67 ± 1.15; 7.33 ± 0.58). Trypanosomosis (4.33–5.67 ± 0.58), tetanus (4.33–6.00 ± 0.58–1.00), and mange (4.33–5.33 ± 0.56–1.73) show year‐round occurrence. In contrast, anthrax is strongly dry season related, peaking in Jiilaal (15.67 ± 2.00) (Table 4).

**TABLE 4:** Matrix scoring for the seasonal occurrence of common camel diseases (mean score for seasons, mean ± SD).

Disease (local name)	*W*	*χ* ^2^ (df = 3)	*p* value	Gu/Spring (Mar–Jun)—rain	Xagaa/Summer (Jun–Aug)—dry	Dayr/Autumn (Sep–Nov)—rain	Jiilaal/Winter (Dec–Feb)—dry
Trypanosomosis (Dhukaan)	0.57	5.12	0.16	5.33 ± 0.58	4.33 ± 0.58	5.67 ± 0.58	4.67 ± 0.58
Anthrax (Kud)	0.81	7.32	0.06	2 ± 0	1.67 ± 1.5	0.67 ± 1.15	15.67 ± 2.0
Wry neck (Shimbir)	0.88	7.88	0.049^∗^	3.67 ± 0.58	10 ± 2	3 ± 1	3.33 ± 0.58
Tetanus (Taagow)	0.61	5.50	0.14	4.33 ± 0.58	5 ± 0	4.67 ± 1	6 ± 0.58
Pneumonia (Dhugato)	0.72	6.52	0.09	11.67 ± 2	2 ± 1	3.33 ± 2	3 ± 2
Mange (Cadho)	0.41	3.66	0.30	5.33 ± 0.58	4.33 ± 0.58	5.33 ± 0.58	5 ± 1.73
Camelpox (Furuq)	0.97	8.77	0.033^∗^	8.67 ± 1.15	0.67 ± 0.58	8.67 ± 1.15	2 ± 1.73
Mastitis (Candho barar)	0.949	8.54	0.036^∗^	7.67 ± 0.58	2.67 ± 0.58	7.33 ± 0.58	2.33 ± 0.58

*Note: k* = 3 informant groups. *W*: Kendall’s coefficient of concordance (*W* = 1 indicates perfect agreement among groups; values closer to 1 indicate stronger concordance). Scoring was performed out of 20 counters distributed across the four seasons for each disease; a higher score means pastoralists more strongly associated that disease with the given season. In Somalia, Gu and Dayr are the rainy seasons; Xagaa and Jiilaal are the dry seasons.

^∗^Marks agreement that is statistically significant at *p* < 0.05.

Statistically significant seasonal differences were observed for wry neck (*χ* ^2^ = 7.88, *p* = 0.049), camelpox (*χ* ^2^ = 8.77, *p* = 0.033), and mastitis (*χ* ^2^ = 8.54, *p* = 0.036), while other diseases showed nonsignificant variation (*p* > 0.05). Overall, results confirm strong climatic influence on disease distribution, with higher burden during wet seasons and anthrax clustering in dry periods (Table 4).

Spearman’s rank correlation was applied to examine the association between mean rank and SSI frequency. The results indicate a weak positive relationship (*ρ* = 0.313), but this association is not statistically significant (*p* = 0.451). The analysis is based on a small sample (*N* = 8), which may limit the statistical power available to detect a true correlation (Table 5).

**TABLE 5:** Spearman’s rank‐order correlation between mean rank and SSI disease frequency.

Variables	Spearman’s rho (*ρ*)	*p* value	Sample size (*N*)
Mean rank vs. SSI disease frequency	0.313	0.451	8

## 4. Discussion

Using thematic analysis of the key informant interviews, the study revealed a variety of camel diseases prevalent in the Banadir and Lower Shabelle regions. Trypanosomosis and mastitis were the most frequently mentioned diseases, signaling their significant perceived effects on camel health and productivity.

This finding is supported by previous studies, which reported mastitis in the Banadir region [20–22] and trypanosomosis [9, 23]. The findings highlight the endemicity of trypanosomosis and mastitis and their widespread impact in the study area.

Other diseases reported by the informants also align with available literature. For instance, mange was reported in camels in the Banadir region [24] and in the Fafan zone eastern Ethiopia [25]. Ringworm [26], hemorrhagic septicemia [12], and anthrax [8] were reported previously from Banadir and Middle Shabelle. Similarly, camelpox, helminthiasis, pneumonia, and tick infestation were previously reported in camels [10, 14, 27, 28]. These strongly aligned studies support the informants’ observations, reinforcing that the identified diseases are indeed prevalent and pose major health challenges for camels in the study area, which highlights the necessity to enhance veterinary services, disease surveillance, and education campaigns to mitigate the impact of these diseases on camel populations.

Key informant interviews also revealed that management of camel disease among farmers predominantly relies on informal methods. Ten of the 17 informants indicated that farmers depend on personal experience and the advice of peers rather than seeking veterinary expertise, largely because of limited awareness and a poor perception of professional services, and 13 of the 17 reported that farmers rely on daily self‐observation of animal health while systematic, regular checkups are uncommon. For instance, over 90% of livestock owners in southwest Nigeria were reported avoidance from veterinary consultation [29], and in Somalia, Middle Shabelle region farmers stated they manage livestock health independently on their own [8]. Similarly, in Borana, Ethiopia, around 90% of owners mentioned they treat their animals themselves using modern drugs and herbal remedies by traditional healers [30]. This reflects a prevalent dependence of farmers on themselves without consulting veterinarians, which is likely to be jointly produced by awareness and perception gaps on the demand side and by genuine access, cost, and reliability constraints on the supply side, making a strong case for community‐based animal health workers and mobile vet services in the region [31]. Widespread unsupervised self‐medication with modern drugs including antibiotics reported in the study could lead to incorrect dosing, milk and meat residues, and antimicrobial resistance as reported elsewhere [32]. This makes it a food safety and public health concern as much as an animal health one, given the central place of raw camel milk in the Somali diet.

Interview results revealed a variety of disease management practices in the study area including use of modern medicine, traditional remedies, and isolation. However, preventive measures such as vaccination and hygiene were less emphasized. Traditional methods mostly reported by informants include burning, administering goat soup, *Dobera glabra* (Garas) seeds, *aloe vera*, and camel hump ghee. Less commonly reported practices include bloodletting, fasting, and Quranic recitations for the animals.

These findings are consistent with other previous studies, which reported similar practices in Pakistan, such as cauterization with red‐hot tools, soup from boiled sheep meat, and ash‐based treatments. Plant extracts, Muslim prayers, and smoke to repel flies were also commonly reported [33]. Similarly, Tuareg camel herders in Niger were reported to use traditional methods such as ghee for topical treatments and bloodletting [34]. These findings underline the strong role of traditional practices in camel disease management, shaped by cultural norms, accessibility issues, and economic constraints. The specific remedies reported here, particularly *Dobera glabra* seeds, *aloe vera*, and camel hump ghee, reflect the ethnobotanical knowledge of the Somali pastoral community and merit documentation both as cultural heritage and as leads for further pharmacological study.

Regarding impacts of diseases, informants emphasized reduced milk production, reproductive issues, animal mortality, market losses, weight loss, growth retardation, and high treatment costs as the main consequences of camel diseases. Psychological effects, including owner aggression and mental health challenges, were also reported.

These findings are consistent with regional observations. In Puntland, Somalia, conditions such as mastitis and endoparasites were identified as key constraints on milk production [22]. In the Somali region of Ethiopia, disease outbreaks caused a 49% and 46% decline in camel milk prices in Babile and Gursum Woredas, respectively, coupled with reduced milk (92%) and meat (78%) demand, as well as plummeting live camel prices [35]. Similarly, in Western Uganda, economic losses due to animal depreciation, high treatment costs, and market closures were reported [36].

These cross‐regional findings demonstrate that camel diseases have far‐reaching economic, nutritional, and public health consequences, highlighting the need for comprehensive and context‐specific disease prevention and control strategies. This is particularly important in Somalia, where camels play a central role in pastoral livelihoods and household resilience. To cope with these impacts, farmers reportedly sold healthy stock to generate income, diversified into other businesses or drew on other livestock species they kept, sought loans or assistance from relatives and friends, and moved animals onto communal rangeland to reduce feeding costs. Several of these responses are, however, double‐edged: selling healthy breeding stock to meet the costs of disease erodes the herd’s future productivity, and concentrating on shared rangeland can itself facilitate disease transmission [31]. This suggests that disease control and livelihood support need to be planned together rather than in isolation.

Informants also highlighted several significant challenges faced by camel farmers, including diseases, inadequate veterinary services, high feed costs, limited knowledge, and insufficient infrastructure such as poor roads and housing. Broader socioeconomic and environmental issues, including insecurity and poor government support, further exacerbate these challenges. These findings are consistent with challenges reported in other regions including camel diseases particularly mastitis, swollen abdomen, and ecto‐ and endoparasites [37]. The surveys also reported feed shortages and poor grazing conditions as key issues [37]. Similarly, the GEEL project conducted a survey in the Waqooyi‐Galbeed region of Somaliland, with 84.2% of respondents revealing feed shortages and droughts as primary concerns, 24.1% citing camel diseases, and 11.6% mentioning pasture depletion.

Another study carried out in southern Ethiopia reported the main constraints impacting camel production including, in order of significance, diseases, feed and water scarcity, inadequate veterinary services, and limited attention to camels’ health [38]. Another study in Borana, Ethiopia, reported the major constraints to camel production to be widespread diseases (25%), lack of attention to camels (20%), inadequate veterinary services (21%), and feed shortage (12%) [30]. In Garissa and Isiolo of Kenya, disease was reported as the most important constraint followed by feed shortage, theft, and marketing [39]. Similarly, in the Banadir region, high feed cost (40%) was reported as the primary concern, followed by poor knowledge (27%), disease (24%), and poor market access (9%) [40]. The convergence of findings across these regions underscores the systemic nature of these challenges, while the prominence of conflict, insecurity, and weak public institutions reflects the specific Somali context, in which prolonged instability has eroded formal veterinary infrastructure.

Eight of the most identified camel diseases in the area were selected for pairwise ranking. The results revealed that anthrax, locally known as “Kud,” was perceived as the most severe disease, with a high mean severity score of 6.67 ± 0.58. The high score reflects a widespread acknowledgment of its potential to cause devastating impacts on camel health and livelihoods. This finding is consistent with evidence from Lower Jubba, Somalia, where camel keepers frequently ranked anthrax among the four most severe camel diseases [23].

Tetanus, locally known as Taagow, was ranked as the second in terms of severity with a notable mean severity score of 6.00 ± 1.00, underlining its significance as a critical health concern. Its high ranking may be related to the distinctive and alarming clinical presentation recognized by camel keepers, including stiffness and paralysis, together with its perceived poor response to treatment. Camel herders in Lower Jubba similarly identified tetanus as one of the most critical camel diseases [23].

Wry neck (Shimbir) is ranked as the third most severe camel disease in this study, with a moderate mean severity score of 4.33 ± 1.15, which is comparable with the findings from Lower Jubba, which ranked the disease as the second most severe [23]. Its relatively high position may be explained by its visible neurological manifestations and poor perceived prognosis. Camels developing the characteristic twisted‐neck posture are reported to recover less frequently than animals experiencing temporary recumbency without neck deviation [30].

Trypanosomosis, locally known as Dhukaan, is ranked fourth in this study, with a similarly moderate mean severity score of 4.00 ± 0.00. This finding aligns with the previous observations [23]. Although it was among the most frequently reported diseases, it was ranked below the acute and rapidly fatal conditions. This distinction indicates that informants did not simply equate frequency with severity. Instead, their rankings appear to reflect differences in disease progression, mortality, treatment possibilities, and livelihood consequences.

Pneumonia (Dhugato) is ranked fifth in this study, indicating a moderate to low severity level. A comparable respiratory condition, chronic coughing syndrome or Duguda, was ranked seventh among nine camel diseases in southern Ethiopia [38]. Its lower position should not be interpreted as evidence that pneumonia is unimportant. Rather, camel keepers may perceive it as relatively more treatable, less uniformly fatal, or slower in progression than anthrax, tetanus, and wry neck.

Diseases such as mange (Cadho) and mastitis (Candho barar) receive lower severity scores and ranks, suggesting lesser concern. These conditions are generally chronic and production‐limiting rather than immediately fatal, which may explain why they were ranked below acute diseases. Therefore, the lower ranking could be interpreted as lower perceived immediate lethality rather than an absence of livelihood importance.

Camelpox was viewed as the least severe with the lowest score, which should not be read as low importance; camelpox is frequently endemic and highly transmissible, and its modest severity score could reflect the largely self‐limiting course of infection in adult animals rather than an absence of impact at the herd level [30].

The disease hierarchy is consistent with the wider literature on pastoralist disease prioritization in the Horn of Africa, where acute, high‐mortality, and zoonotic conditions are repeatedly ranked above chronic or production‐limiting ones, and where locally named syndromes map closely onto recognized clinical entities [38, 41, 42] Triangulating the community priorities identified in Table 2 with laboratory confirmation and clinical records for the Banadir and Lower Shabelle regions, and extending the exercise to additional communities, would allow the strong perceptual consensus observed here to be validated against measured disease occurrence and would strengthen the evidence base for camel disease control in Somalia.

Our analysis utilizing matrix scoring demonstrates a substantial convergence between pastoralists’ indigenous knowledge and established veterinary understanding of camel diseases. The strongest associations included nasal discharge and dyspnea with pneumonia, sudden death with anthrax, paralysis and stiffness with tetanus, reduced milk production with mastitis, and progressive wasting and odor change with trypanosomosis. These findings indicate that informants used recognizable clusters of clinical signs to distinguish diseases rather than relying on a single, nonspecific observation.

Notably, dyspnea and nasal discharge emerged as significant indicators of pneumonia, corroborating findings from a FGD in southern Ethiopia, where the majority linked nasal discharge with pneumonia [38]. Additionally, loud, severe coughing with open‐mouth breathing was similarly associated with pneumonia in camels, as observed in previous research [30].

A distinct odor change was predominantly linked to trypanosomosis in our study, echoing previous observations from Baidoa, Somalia, where camels afflicted with trypanosomosis exhibited a strong, pungent odor [43]. Similar studies noted a unique odor in the urine of animals infected with the disease [33]. Although odor is not sufficiently specific to confirm infection, its repeated identification by different studies suggests that it may form part of a broader local diagnostic pattern.

Paralysis and stiffness were consistently attributed to tetanus and wry neck, aligning with findings from Boran, who noted paralysis associated with wry neck [30]. This agreement indicates that the informants clearly recognized neurological dysfunction as an important feature of both conditions.

Fever, a common symptom, was linked with both camelpox and trypanosomosis. This association is widely supported in the literature, indicating a broad understanding among camel farmers regarding the significance of fever as a clinical sign in these diseases [33, 38, 44].

The study further revealed a strong correlation between trypanosomosis and emaciation, likely due to the chronic nature of the disease, a finding supported by other researchers [23, 38]. Additionally, there was a significant association noted between reduced milk production and mastitis. While various febrile infections can reduce milk output, mastitis specifically affects the udder, significantly impacting milk production, as evidenced by a study in Puntland, Somalia [22].

Finally, the critical severity of diseases such as anthrax and tetanus was underscored by their association with sudden death, supported by reports from the Middle Shabelle region, Somalia, where a significant proportion of respondents linked sudden death predominantly with anthrax [12].

On the other hand, indicators that are loaded onto two diseases simultaneously carry a cautionary message for surveillance design. The overlap of paralysis and stiffness between tetanus and wry neck, and of skin lesions between mange and camelpox, indicates that these disease pairs cannot be discriminated on the basis of a single sign and are therefore liable to be confused. Furthermore, several limitations should be noted. The concordance analysis was based on only three informant groups, limiting the number of independent observations despite the strong agreement observed. In addition, the scores reflect perceived disease associations rather than laboratory‐confirmed diagnoses.

The correlation between pastoralists’ disease ranking and SSI‐based frequency was weak and nonsignificant (*ρ* = 0.313, *p* = 0.451, *N* = 8). With only eight diseases, this coefficient cannot be distinguished from chance, so the result reflects limited statistical power rather than true independence. The weak alignment is nonetheless informative: Pastoralists rank diseases by overall impact rather than frequency, so an uncommon but highly fatal disease may outrank a frequent but mild one [38, 45].

The study indicated that camelpox, pneumonia, and mastitis were perceived to occur more frequently during the rainy season, consistent with findings from other camel‐producing areas [30, 33, 38]. The agreement between community observations and published epidemiological evidence suggests that camel keepers possess detailed temporal knowledge based on repeated observation across production cycles. Several interacting factors which can contribute to the rainy season increase in these diseases include poor housing conditions, as camels are often kept in open pens with minimal shade, leaving them exposed to rain and wet environments. Such exposure weakens their immunity, enhances pathogen survival, and increases vector activity, which may contribute to the transmission of certain diseases [46–48]. Furthermore, the rainy season’s heightened camel mobility during migration from dry‐season grazing areas amplifies the risk of disease spread [42, 49].

Anthrax was perceived to occur more frequently during the dry season, in agreement with reports from several previous studies in different locations [50–52]. This is attributed to animals grazing in floodplains and other areas inaccessible during the wet season, where anthrax spores may be exposed. Increased anthrax cases in both cattle and humans are usually linked to this seasonal grazing pattern [51], emphasizing the need for targeted management of grazing areas to reduce outbreaks. Research from the upper Zambezi floodplain similarly demonstrated that anthrax occurrence was strongly shaped by seasonal land use and human and livestock dependence on floodplain resources [51]. The dry‐season pattern reported here should therefore be validated locally rather than assumed to apply uniformly across all ecological settings.

The findings of this study demonstrate that camel mange occurs consistently throughout the year across all seasons. This observation aligns with other studies, which reported a similar seasonal pattern of mange infection rates [30]. This may reflect the direct or close‐contact transmission of mange mites and their continued circulation within affected herds. Although the study did not stratify findings by region or district, the observed year‐round occurrence of mange is consistent with features of intensive and semi‐intensive camel production systems reported in parts of the Banadir region [40]. In these systems, close and repeated contact between animals, confinement in shared enclosures, and the frequent introduction of susceptible camels may support continued transmission throughout the year, even when rainfall‐related influences are minimal. This may explain the difference from studies reporting higher mange prevalence during hot or rainy months [53].

Trypanosomosis was also perceived to occur throughout the year. This observation is biologically plausible because the principal camel trypanosome (Trypanosoma evansi) is mechanically transmitted by several groups of biting flies rather than being dependent exclusively on tsetse flies [9, 38]. Studies in southern Ethiopia have similarly reported infection across different seasons, although prevalence and vector abundance may vary during the year [38]. In contrast, other studies observed higher prevalence during the rainy season, attributing this to increased vector activity [54]. A possible explanation for this could be that year‐round community recognition does not necessarily imply that incidence is constant throughout the year. Chronic infections may remain clinically apparent for long periods, while seasonal increases in biting‐fly abundance may still produce peaks in new infections. Longitudinal parasitological and entomological investigations would be required to distinguish persistent clinical disease from seasonal transmission.

Overall, the community‐derived seasonal calendars demonstrate well‐developed knowledge of relationships among rainfall, animal movement, herd management, vector activity, and disease occurrence. The broad correspondence with independent epidemiological studies is encouraging and supports the use of pastoralist knowledge to guide the timing of surveillance, vaccination, vector control, community education, and mobile veterinary services. Nevertheless, perceived seasonality should be triangulated with longitudinal clinical records, laboratory testing, meteorological information, and vector surveillance. Participatory and conventional epidemiological methods are most informative when used as complementary sources of evidence rather than as substitutes for one another [31, 45].

## 5. Conclusion and Recommendations

This study documented community‐perceived epidemiology and management of common camel diseases in the Banadir and Lower Shabelle regions of Somalia. Pastoralists identified mastitis, trypanosomosis, mange, pneumonia, and camelpox as the most frequently encountered conditions and ranked anthrax and tetanus as the most severe, chiefly because of their high case fatality and association with sudden death.

Across the three informant groups, matrix scoring showed strong and statistically significant agreement in linking clinical signs to specific diseases—for example, dyspnea and nasal discharge with pneumonia, emaciation, and odor change with trypanosomosis, paralysis and stiffness with tetanus, and reduced milk yield with mastitis. This concordance reflects a consistent and substantial body of indigenous knowledge; it should be interpreted as agreement among informants rather than as diagnostic confirmation, and the perceived sign–disease and seasonal associations (pneumonia, camelpox, and mastitis with the rain; anthrax with the dry season; trypanosomosis, tetanus, and mange year‐round) warrant validation through clinical and laboratory investigation.

Camel diseases were reported to impose substantial impacts including reduced milk yield, reproductive failure, weight loss, market losses, and high treatment costs, which farmers cope with by selling healthy stock, diversifying income sources, and borrowing from relatives and friends. Disease management relied heavily on self‐treatment with limited veterinary input, combining modern drugs with traditional practices such as cauterization, medicinal plants (notably *Dobera glabra* and *aloe vera*), goat soup feeding, and application of camel hump ghee, alongside preventive measures including relocation, isolation, quarantine of affected animals, and Quranic recitation. These practices, together with feed shortages, weak veterinary services, and limited access to quality drugs, define the key constraints on camel health in the study area.

Overall, the study highlights several priorities for the study area: further research into traditional camel disease treatments and their proper documentation; greater education and awareness among camel farmers on the importance of veterinary consultation and the correct use of veterinary drugs; and improved veterinary service delivery and accessibility, supported by subsidies. Affordable vaccines and drugs should be prioritized, and a holistic approach to disease control combined with better feed availability is essential to address the seasonal challenges farmers face.

## Author Contributions

Omar Salah conceptualized and designed the study, coordinated the research project during all stages, and served as the corresponding author.

Yasin Sh Hassan contributed to the development of study methodology and involved in data collection and formal analysis.

Mohamed Yusuf conducted the data collection and analysis and wrote the manuscript.

Hassan AbdiNB involved in validation, reviewing, and editing of the manuscript.

## Funding

No external funding was used during the study; the study was carried out with self‐funding by the authors.

## Ethics Statement

Before the study is carried out, ethical approval was obtained from the Research Ethical Committee of the Faculty of the Veterinary Medicine and Animal Husbandry of Somali National University (Approval No. FVM‐AH/REC/2024/09).

## Conflicts of Interest

The authors declare no conflicts of interest.

## Data Availability

All data analyzed and presented in this study are available upon request from the corresponding author.
